# Focusing on the essentials: learning for performance

**DOI:** 10.1186/1478-4491-6-26

**Published:** 2008-12-10

**Authors:** Catherine J Murphy

**Affiliations:** 1 IntraHealth International, Inc., 6340 Quadrangle Drive, Suite 200, Chapel Hill, NC 27517, USA

## Abstract

As *The World health report 2006 *emphasized, there is increasing consensus that training programmes should focus on "know-how" instead of "know-all." Health workers need to know how to do the job they will be expected to do. IntraHealth International's *Learning for performance: a guide and toolkit for health worker training and education programs *offers a step-by-step, customizable approach designed to develop the right skills linked to job responsibilities. Using *Learning for performance *(*LFP) *yields more efficient training that focuses on what is essential for health workers to do their jobs and on effective learning methods, while addressing the factors that ensure application of new skills on the job.

This brief communication describes the *Learning for performance *approach and initial findings from its application for pre-service education and in-service training in three countries: India, Mali and Bangladesh. Based on IntraHealth's experiences, the author provides thoughts on how *LFP*'s performance-based learning approach can be a useful tool in training scale-up to strengthen human resources for health.

## Background

Training is frequently proposed as a stand-alone intervention to fix a service delivery problem. This use of training often fails to bring about desired changes in health services because the support needed to apply newly learned skills in the work environment is lacking. Too often, training curricula are laden with content that is not related to job responsibilities and do not provide adequate opportunities for practice, thus diluting job-related learning. Delivering bloated curricula takes too much time when health systems have a severe shortage of "the right health workers with the right skills in the right place doing the right thing." [1]. Countries with shortages cannot afford for their existing health workers to be away from service sites for long periods for training, especially training that does not yield results.

*Learning for performance *(*LFP*) [2] is a systematic instructional design process with practical tools developed by IntraHealth International with support from the United States Agency for International Development (USAID). *LFP *can be an important tool for scaling up workforce training and education, because it emphasizes:

• efficiency (by removing unnecessary content and retaining only essential content, thus shortening the time required for training);

• relevance (to the work environment and specific job responsibilities);

• preparing learners for job performance (by using experiential, competence-based training methods and improving pre- and post-training support so that training does not occur in isolation).

**Figure 1:**
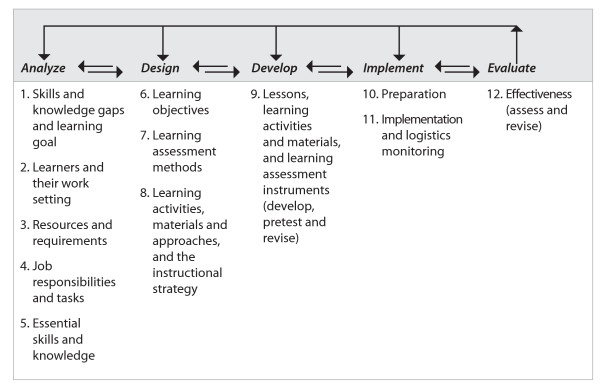
The instructional design process and *Learning for performance *steps.

The 12 steps of *Learning for performance *are clustered under the five phases of instructional design (Fig. 1).

## Discussion

IntraHealth has applied the *Learning for performance *approach in a variety of countries and situations – public sector, private sector, pre-service education, in-service training – and with a range of health worker levels from physicians and nurses/midwives to community-based health workers.

In India, IntraHealth used *Learning for performance *to assist the Ministry of Health in Uttar Pradesh to revise the chapter on postpartum care of the pre-service curriculum for a new health cadre: private community midwives (CMWs). During the revision process, 13 pages of content not related to CMWs' job responsibilities were removed, all content was updated and content was added to strengthen clinical exercises and cover a critical postpartum care responsibility overlooked in the original chapter.

A post-test control group study (Murphy C, Hassett P, Ansingkar A, Srikar P, Singh V: A study to assess the effectiveness of using *Learning for performance *to revise one chapter of the India community midwives' pre-service training curriculum. Chapel Hill, NC: IntraHealth PRIME II Project; 2004, unpublished) evaluated the effectiveness and usability of the *LFP*-revised chapter and found significant improvement. The *LFP*-revised chapter was implemented for 102 CMW trainees in two intervention districts, while the original chapter on postpartum care was implemented for 113 CMW trainees in two control districts. At the intervention sites, 74.3% of students were able to perform overall to standard on clinical skills, compared with only 16.7% of students at the control sites. On the end-of-chapter knowledge test, average scores in the intervention sites ranged from about the same to significantly better than scores from the control sites.

This study concluded that using *Learning for performance *to focus a curriculum on essential content and appropriate performance-based learning methods can yield improved knowledge and skills performance. It cautioned that other factors besides the curriculum design contribute to learning and performance and should also be addressed. These factors include trainer skills and motivation, facilities and equipment, and institutional support.

In Mali's resource-poor northern zone, IntraHealth used *Learning for performance *to assist the Ecole des Infirmiers de Gao (EIG) develop the family planning/reproductive health (FP/RH) and child health (CH) modules for the nursing and midwifery school curriculum. The school's faculty, clinical trainers and directors learned how to use the *LFP *approach. They visited health facilities that employ the school's graduates to observe and discuss critical FP/RH and CH services needed by the community and the related job tasks and facility improvements to provide these services. They then revised the learning objectives, content and evaluation methods to address these job tasks, ensuring that graduates could provide services that the communities needed. Faculty introduced interactive, performance-based learning methods into their teaching and began work on five additional modules using the *LFP *process.

The FP/RH module is being introduced into the curriculum in 2008, and its implementation will be monitored and evaluated. Student performance on national exams will be compared with previous years' student performance. In the meantime, the school directors have already noted several positive outcomes [3]. EIG's Director of Studies stated that "before using *Learning for performance*, each faculty member determined his own content to cover, which led to wide variations of a module from one year to the next and from one faculty member to another. The performance-based approach will enable the school to standardize the curriculum with an emphasis on meeting the competency needs of the students." He added that "performance-based learning significantly reduces the gap between continued education and the base curriculum. For example, family planning is now taught at the school in all its components whereas before, students learned once they were in the field."

In Bangladesh, IntraHealth used *LFP *to assist USAID's NGO Service Delivery Project to adapt the group-based national family planning in-service training curriculum to an on-the-job training (OJT) approach (Murphy C, Meena U: NSDP's decentralized training strategy. Dhaka: NGO Service Delivery Program; 2007, unpublished). Bangladesh's network of nongovernmental organizations (NGOs) that provide health services according to the government's essential services package decided to pilot an OJT approach to updating their new employees' FP skills to avoid the costs and service interruption of sending staff to a residential training programme in the capital city.

The NGOs developed OJT courses in counseling skills and infection prevention. During the pilot test of the courses, trainees learned essential job-related content and participated in simulated and actual client practice as often as needed to develop their counseling and clinical skills. They were not limited by the set schedule and lack of access to clients that hampers traditional group-based training.

In a post-test assessment of knowledge and competency, all trainees (n = 18) passed the knowledge tests and achieved competence in the skills assessment. The NGO directors appreciated that OJT trainees could proceed at their own rate, studying and practising during slow clinic hours, and not interrupting services. After the pilot test, the NGOs used *LFP *to develop a third OJT course, on intrauterine device (IUD) services, and all three courses are being scaled up through a decentralized training approach.

## Conclusion

Because of its flexible approach applicable to pre-service education or continuing education and in-service training, and its focus on essential content, skills and knowledge while delivering specific job-related outcomes, *Learning for performance *can be a useful approach in strategies to develop the health workforce, through:

• scaling up or accelerating training in order to quickly increase sheer numbers of competent health providers;

• adding or shifting job tasks among health providers;

• efficiently developing new health cadres;

• upgrading health worker skills so that they can advance to positions in higher priority cadres;

• ensuring that students graduating from pre-service education programmes are ready to start providing essential health services without needing additional in-service training.

Using *LFP *yields training that concentrates on what is essential for health workers to do their jobs and on effective learning methods while addressing the factors in the learning and work environment that ensure application of new skills on the job. These qualities make *LFP *a practical and results-driven tool for scaling up training to strengthen human resources for health.

## Competing interests

The author declares that she has no competing interests.
